# Typical CT and MRI Features of Mucinous Rectal Adenocarcinoma

**DOI:** 10.5334/jbsr.1910

**Published:** 2019-10-04

**Authors:** Jin-A Ryoo, Seung Soo Kim

**Affiliations:** 1 Department of Radiology, Soonchunhyang University College of Medicine, Cheonan Hospital, Cheonan-si, KR

**Keywords:** Rectal neoplasms, Mucinous adenocarcinoma, Computed tomography, Magnetic resonance imaging

## Abstract

**Main teaching point:** Mucinous rectal adenocarcinoma typically shows high signal intensity on T2-weighted images, weak enhancement, and internal calcification.

## Case History

**Figure 1:**
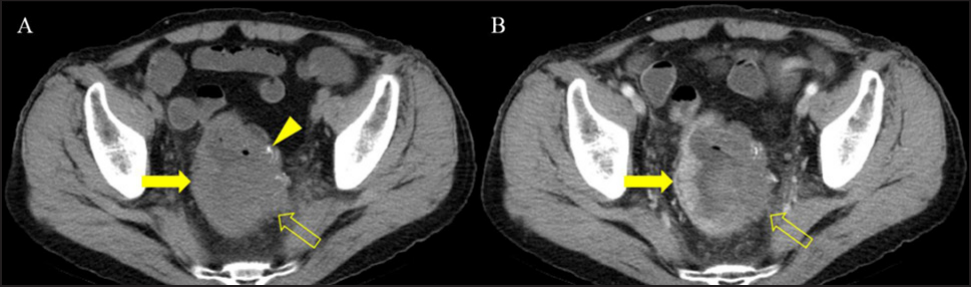


**Figure 2:**
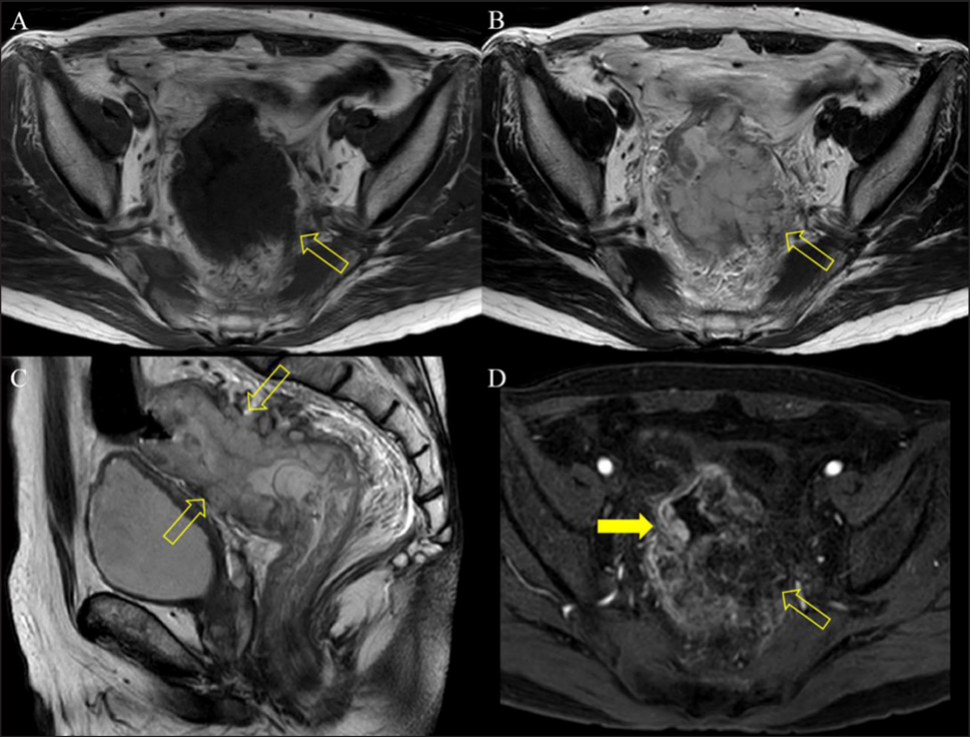


**Figure 3:**
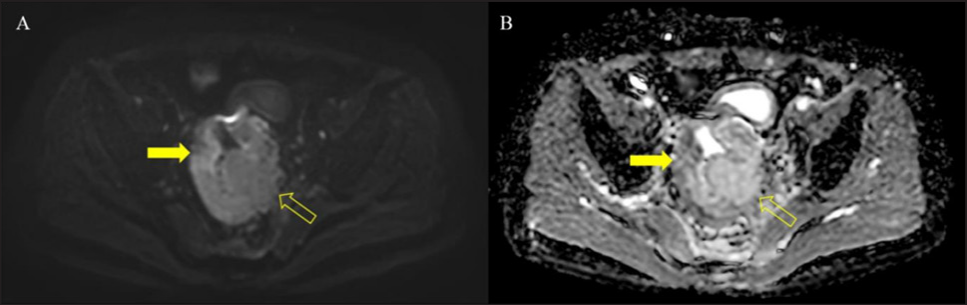


A 69-year-old man was referred to our hospital for evaluation of a rectal mass that was incidentally detected at a local clinic. The patient was diagnosed with adenocarcinoma through colonoscopic biopsy, and computed tomography (CT) and magnetic resonance imaging (MRI) were performed for staging. Axial unenhanced and portal venous phase CT images (Figure 1) showed irregular and severe wall thickening (arrow and open arrow) in the rectum. The left side (open arrow) of the rectum contained calcification (arrowhead) and revealed poor enhancement relative to the right side (arrow). The majority of the wall thickening (open arrows) showed low and high signal intensity on T1- and T2-weighted imaging (Figure 2A–C), respectively. Gadolinium-enhanced MRI (Figure 2D) demonstrated greater enhancement of the right side rectal wall (arrow) compared with the left side wall (open arrow). On diffusion-weighted imaging (b = 800 s/mm^2^) and apparent diffusion coefficient map (Figure 3), the well-enhanced right side rectal wall (arrow) revealed diffusion restriction, while there was no diffusion restriction in the left side wall (open arrow). The patient underwent Hartmann’s operation after concurrent chemoradiotherapy, and the final diagnosis was mucinous rectal adenocarcinoma.

## Comment

Mucinous rectal adenocarcinoma is a distinct pathologic subtype of rectal cancer. The World Health Organization defines mucinous adenocarcinoma as a neoplasm composed of greater than 50% extracellular mucin, which can be pathologically confirmed after surgical resection. Mucinous carcinoma has higher local recurrence, distant metastasis, lymph node metastasis, and venous invasion compared with nonmucinous carcinoma. Thus, a more aggressive surgical approach, including wide excision and extensive lymph node dissection, is needed in patients with mucinous rectal adenocarcinoma than in patients with nonmucinous adenocarcinoma [1].

The abundant extracellular mucin of mucinous carcinoma results in typical CT and MRI features that are different from those observed in nonmucinous carcinoma. Mucinous carcinomas show less enhancement and more severe wall thickening in imaging compared with nonmucinous carcinomas. Moreover, calcium deposition within the wall thickening is the characteristic CT finding of mucinous adenocarcinoma. Extracellular mucin is hyperintense on T2-weighted images, and the presence of a T2-hyperintense signal greater than 50% of tumor volume suggests mucinous adenocarcinoma. Unlike nonmucinous components with diffusion restriction, the mucin components of mucinous adenocarcinoma do not exhibit diffusion restriction [1].
